# Validated Administrative Data-Based ICD-10 Algorithms for Chronic Conditions

**DOI:** 10.23889/ijpds.v9i5.2878

**Published:** 2024-09-18

**Authors:** Angela Kuang, Claire Xu, Danielle Southern, Namneet Sandhu, Hude Quan

**Affiliations:** 1 University of Calgary

## Objective

This systematic review aimed to identify ICD-10 based validated algorithms for chronic conditions using health administrative data.

## Methods

A comprehensive systematic literature search using Ovid MEDLINE, Embase, PsycINFO, Web of Science and CINAHL was performed to identify studies, published between 1983 and May 2023, on validated algorithms for chronic conditions using administrative health data. Two reviewers independently screened titles and abstracts and reviewed full text of selected studies to complete data extraction. A third reviewer resolved conflicts arising at the screening or study selection stages. The primary outcome was validated studies of ICD-10 based algorithms with both sensitivity and PPV of ≥70%. Studies with either sensitivity or PPV <70% were included as secondary outcomes.

## Results

Overall, the search identified 1686 studies of which 55 met the inclusion criteria. Combining a previously published literature search, a total of 61 studies were included for data extraction. Of these, 30 studies demonstrated algorithms with high validity and 30 showed moderate validity. Studies with highly valid algorithms were based on administrative data from different countries including Canada, USA, Australia, Japan, France, South Korea, and Taiwan. Validated algorithms with PPV and sensitivity of ≥70% were identified for more than 40 chronic conditions that included several types of cancers, cardiovascular conditions, kidney diseases, gastrointestinal disorders, and peripheral vascular diseases, amongst others.

## Conclusion

With ICD-10 prominently used across the world, this up-to-date systematic review can prove to be a helpful resource for research and surveillance initiatives using administrative health data for identifying chronic conditions.

